# Cardiac Shockwave Therapy – A Novel Therapy for Ischemic Cardiomyopathy?

**DOI:** 10.3389/fcvm.2022.875965

**Published:** 2022-05-12

**Authors:** Michael Graber, Felix Nägele, Jakob Hirsch, Leo Pölzl, Victor Schweiger, Sophia Lechner, Michael Grimm, John P. Cooke, Can Gollmann-Tepeköylü, Johannes Holfeld

**Affiliations:** ^1^Department of Cardiac Surgery, Medical University of Innsbruck, Innsbruck, Austria; ^2^Department of Cardiovascular Sciences, Center for Cardiovascular Regeneration, Houston Methodist Research Institute, Houston, TX, United States; ^3^Division of Clinical and Functional Anatomy, Medical University of Innsbruck, Innsbruck, Austria; ^4^Department of Cardiology, University Hospital Zurich, Zurich, Switzerland

**Keywords:** shockwaves, angiogenesis, regeneration, ischemic heart disease, translational research

## Abstract

Over the past decades, shockwave therapy (SWT) has gained increasing interest as a therapeutic approach for regenerative medicine applications, such as healing of bone fractures and wounds. More recently, pre-clinical studies have elucidated potential mechanisms for the regenerative effects of SWT in myocardial ischemia. The mechanical stimulus of SWT may induce regenerative effects in ischemic tissue *via* growth factor release, modulation of inflammatory response, and angiogenesis. Activation of the innate immune system and stimulation of purinergic receptors by SWT appears to enhance vascularization and regeneration of injured tissue with functional improvement. Intriguingly, small single center studies suggest that SWT may improve angina, exercise tolerance, and hemodynamics in patients with ischemic heart disease. Thus, SWT may represent a promising technology to induce cardiac protection or repair in patients with ischemic heart disease.

## Background

Ischemic heart disease (IHD) remains the most frequent cause of death in the Western World (1). IHD can result in necrotic death of cardiomyocytes and their subsequent replacement by non-functional scar tissue (2). Contractile function of the scarred and ischemic myocardium is impaired in ischemic cardiomyopathy (ICMP). One strategy to preserve myocardial tissue is myocardial protection. For example, myocardial protection may be achieved during cardiac surgery by applying cold cardioplegic solution to decrease myocardial oxygen consumption and thus, avoid myocardial damage during ischemia. Remodeling of the heart is an alteration in the dimensions of the ventricular wall and/or chambers. Correction of myocardial ischemia can lead to reduced left ventricle chamber volume. Myocardial regeneration is achieved when new myocardial cells (cardiomyocytes, endothelial and/or vascular smooth muscle cells) are generated from progenitor cells or proliferation of resident cardiac cells. The optimal management of ICMP would restore perfusion, increase proliferation and function of cardiac cells, to improve ventricular function and structure.

One way of improving heart function is the re-establishment of adequate blood supply to perfuse the chronically ischemic border zone recruiting hibernating myocardium (3). Surgical or interventional revascularization is limited to large coronary vessels, and a microvascular deficit may remain.

Angiogenic and regenerative treatment options may address this deficit. Cardiac shockwave therapy (SWT) has had promising effects in small clinical trials, and pre-clinical studies indicate that this benefit may be due to angiogenic, vasculogenic, and tissue regenerative responses (4–7). Several studies over the past years have repeatedly confirmed the angiogenic and regenerative effects of SWT in cell culture and various animal models, including hind limb ischemia and acute or chronic myocardial ischemia (8–11). In parallel, clinical studies investigating cardiac SWT have observed symptomatic relief in patients with refractory angina (5, 12, 13) as well as improvement of left ventricular function in patients with ICMP (14–16) indicating its promise in clinical use.

This review summarizes our present knowledge on this promising technology and addresses gaps of knowledge that have yet to be answered in future trials.

## Safety Aspects

Shockwaves are specific sound-pressure waves appearing as transient pressure oscillations with characteristic wave profiles. The specific features defining the different types of shockwaves and the four technologies currently available to produce them have been discussed in previous reviews (17, 18). Notably, only focused shockwaves are used in the context of heart failure therapy. Shockwaves were originally applied for the purpose of lithotripsy to disintegrate kidney and urethral stones (19). As an incidental finding, iliac bone thickening was observed upon SWT. This serendipitous observation led to studies to assess SWT for bone regeneration in patients with non-unions and bone defects (20). Subsequent studies revealed that SWT could enhance healing of soft tissue defects or non-healing wounds (21, 22).

The observed regenerative effects were mainly attributed to inducing micro-injuries to the tissue, followed by subsequent repair. However, studies published over the recent years clearly showed a beneficial effect of SWT even at lower energies. Thorough examinations of tissues after SWT were not able to detect any signs of cellular damage. Transmission electron microscopy analyses of hearts treated with SW showed no changes of the myocardial ultrastructure upon therapy (7). Treatment of ischemic hearts in large animal models resulted in no signs of arrhythmia or functional impairment (23). A recent paper provided evidence for a therapeutic range of SWT, showing no cellular damage of cardiac cells beneath energy levels of 0.27 mJ/mm^2^ total flux density. Regenerative effects including endothelial cell proliferation and angiogenic gene expression are induced dose-dependently until 0.15 mJ/mm^2^ energy flux density. *In vitro* studies to characterize the effects of SWT revealed that in addition to the intensity of shockwaves, the effects of SWT were influenced by the geometry of the cell culture flask due to physical phenomena including reflection and interference (24). Moreover, the number of impulses has an impact on cell viability (25). However, there is no evidence that SWT induces cellular damage when used within a therapeutic range.

## Proliferation

One crucial mechanism underlying the regenerative effect of SWT is the induction of cellular proliferation. With respect to the heart, this proliferative effect was described mainly for endothelial cells. Although SWT induces proliferation of fibroblast cell lines *in vitro* possibly *via* transforming growth factor beta (TGF-β) upregulation (25), there is no evidence of proliferation of cardiac fibroblasts upon SWT *in vivo* (24). As cardiomyocytes are post-mitotic cells with very limited capacity of proliferation, it seems very unlikely that SWT might cause proliferation in primary cardiac myocytes. Indeed, *in vitro* studies of a cardiomyocyte cell line showed no proliferative effects of SWT upon treatment, irrespective of treatment dose (24).

There is ample evidence that SWT induces proliferation of endothelial cells (26). This might be due to the release of vascular endothelial growth factor (VEGF) and activation of VEGF receptor 2 (VEGFR2) with subsequent activation of AKT/ERK pathways resulting in endothelial cell proliferation (growth factor release upon SWT is discussed below). Interestingly, the proliferative effects of SWT are abolished upon inhibition of VEGF or VEGFR2 (27). Moreover, proliferation of endothelial cells upon SWT was described *in vivo* after induction of hind limb ischemia (28).

## Pro-Survival/Anti-Apoptotic

Myocardial hypoxia causes loss of cardiac cells. Tissue necrosis within the ischemic core is accompanied by apoptosis of cells in adjacent cardiac tissue, especially in the border zone of infarcted areas (29). At the same time, the evolutionary conserved process of autophagy is initiated, recycling damaged cellular components (30). Autophagy allows cells to adapt to various environmental stresses *via* degradation of defective proteins or organelles by lysosomes. Post-mitotic cells rely on autophagic processes upon stress since cell replacement is not an option (31). In rat cardiomyocytes, SWT promotes autophagy after hypoxia, probably *via* regulation of mammalian target of rapamycin (mTOR) and subsequent activation of AMP-activated kinase (AMPK) and Beclin 1 (32).

**FIGURE 1:**
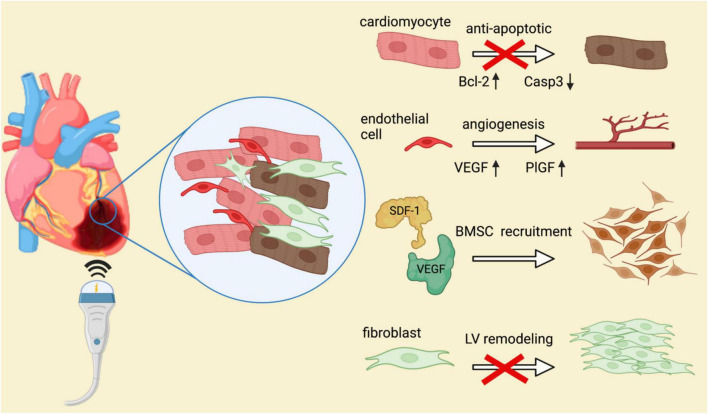
Molecular mechanisms of shockwave therapy in ischemic heart disease. Several mechanisms of positive impacts mediated by SWT on the course of ischemic heart disease have been described. By regulation of anti-apoptotic proteins, cardiomyocyte survival is enhanced and hence, cardiac damage limited. Angiogenesis upon shockwave therapy is mediated *via* release of endothelial growth factors VEGF and PlGF. SDF-1 acts as a chemoattractant of angiogenic bone marrow derived stem cells. Migration of these progenitor cells is crucial for induction of vasculogenesis and revascularization of ischemic tissue. Moreover, shockwave therapy was shown to reduce the number of fibrocytes within chronic ischemic myocardium, thereby promoting reverse LV remodeling.

The limitation of cell death upon infarction is a valuable therapeutic strategy to preserve cardiac function after ischemia (33). SWT inhibits apoptosis in a myocardial cell line upon *in vitro* hypoxia and increases cell viability, thereby having a protective rather than regenerative effect on cardiomyocytes. It increases the expression of the crucial anti-apoptotic protein Bcl-2 and decreases the expression of the pro-apoptotic protein Bax. This effect reduces the activation of Caspase 3, a crucial mediator of the intrinsic pathway of apoptosis. The anti-apoptotic effects might depend on phosphorylation of AKT (34) (Figure 1).

## Growth Factor Release

The release of growth factors is crucial for successful regeneration. Growth factors are tissue-specific proteins with pivotal roles in both development and healing. The subtype of released growth factor upon SWT depends on the treated tissue and pathology. Regenerative effects of SWT in musculoskeletal disorders are mediated by released TGF-β, insulin-like growth factor 1 (IGF-1) and bone morphogenetic proteins (BMPs) (35, 36). These growth factors regulate proliferation of mesenchymal cells, thereby mediating bone, cartilage, and tendon repair. On the other hand, healing of chronic wounds depends on different factors, as these wounds are associated with persistent inflammatory dysregulation. SWT has beneficial effects on the healing of chronic wounds by modulating the inflammatory response. The release of platelet-derived growth factor (PDGF) modulates macrophage recruitment and function and thus contributes to wound healing (37). Macrophages play a prominent role in wound healing by creating granulation tissue, protecting from infection, and facilitating re-epithelization (38). Besides modulation of inflammation, induction of angiogenesis is the key for successful wound healing. Angiogenic growth factors such as VEGF and fibroblast growth factor (FGF) are major determinants of microvessel formation (27, 39). In ischemic tissue, including the heart, SWT induces the release of angiogenic growth factors including VEGF, placenta growth factor (PlGF) and FGF (26, 27, 40). These growth factors might be stored in the extracellular matrix and released upon mechanical stimulus (27).

## Angiogenesis

Angiogenesis is a vital part in regeneration of ischemic tissue. It improves perfusion preventing further ischemic damage and restores tissue function. Angiogenesis is defined as the formation of new capillaries from pre-existing vessels. This process is initiated by angiogenic growth factors driving the sprouting and proliferation of endothelial cells. The most prominent and angiogenic factor is VEGF. VEGF appears in four isoforms, VEGF-A, VEGF-B, VEGF-C, and VEGF-D (41). These peptides bind to and activate their receptors VEGFR1, VEGFR2, and VEGFR3. VEGFR3 is activated by VEGF-C and VEGF-D and generally limited to lymphatic endothelial cells. VEGFR2 binds the most abundant form VEGF-A and facilitates endothelial cell proliferation, migration and survival. Activation of VEGFR1 by VEGF-B and PlGF, another member of the VEGF-subfamily, leads to monocyte recruitment rather than induction of angiogenesis (41). Hence, decisive angiogenic mechanism are depending on VEGFR2 activation. SWT induces VEGF release and subsequent VEGFR2 activation in endothelial cells *in vitro*, resulting in endothelial cell proliferation (42). Moreover, SWT promotes the sprouting of new vessels from *ex vivo* cultured aortic rings (40). In this assay, the same molecular mechanisms are observed.

Similarly, SWT induces angiogenesis in a variety of animal models and tissues. Shockwaves enhance blood flow in epigastric skin flaps and hence, improves skin flap survival. In this case, the increase in microvascular density is associated with the generation of VEGF and nitric oxide (NO) (43, 44). NO is synthesized by endothelial nitric oxide synthase (eNOS), a direct downstream target of VEGFR2-signaling. NO is a potent vasodilator, which also regulates endothelial cell growth and cellular homeostasis (45). SWT similarly improves limb perfusion and function in a hind limb ischemia model in rodents, an effect which is associated with an increase in VEGF and VEGFR2 activation. The treatment increases the number of endothelial cells and capillaries in the ischemic limb musculature (28). Similar results are obtained in the ischemic heart. Shockwave therapy enhances capillary density in the border zones of experimental myocardial infarction, resulting in decreased infarct size and hence, improved cardiac function (27). In addition to angiogenesis, the increase in capillary density might also be due to vasculogenesis, the process by which circulating progenitor cells contribute to the microvasculature. Of interest, shockwave-treated hearts show a greater number of arterioles within the ischemic myocardium, indicative of arteriogenesis, which is the positive remodeling of existing collateral channels.

## Progenitor Cells

Circulating progenitor cells may play a role in revascularization. Such circulating cells may be capable of differentiation toward mature endothelial cells and participate directly in the formation of new vessels (46). On the other hand, other circulating progenitor cells may act in a paracrine fashion by releasing growth factors and creating an angiogenic milieu. Physiologically, endothelial progenitor cells (EPCs) and mesenchymal stem cells (MSCs) are involved in revascularization of ischemic tissue. EPCs are capable of both differentiation toward endothelial cells and release of growth factors (46). Only a small subset of EPCs is of true endothelial lineage in humans, most being of hematopoietic lineage. The great majority of these circulating angiogenic cells promote angiogenesis by secreting angiogenic cytokines and matrix metalloproteinases (47, 48). Some circulating cells that contribute to angiogenesis may be derived from mature endothelial cells from other sites that are mobilized into the systemic circulation by angiogenic cytokines released from the ischemic tissue (49). In addition, resident tissue MSCs may differentiate into pericytes stabilizing the endothelial network and supporting blood vessel growth *via* paracrine secretion (50).

Shockwave therapy may affect progenitor cells in several ways. First of all, SWT induces the release of stromal-derived factor 1 (SDF-1), a chemoattractant and ligand of CXC chemokine receptor 4 (CXCR-4) on EPCs (28, 51, 52). Hence, increased numbers of EPCs migrate to the ischemic tissue and contribute to the process of new vessel formation. Enhanced recruitment of multipotent cells and concomitant vasculogenesis is observed in shockwave-treated ischemic hind limbs as well as in chronic IHD (28, 53). Since SWT improves migration of intrinsic multipotent cells *via* upregulation of chemoattractants, it is also able to induce homing of systemically injected stem cells (52). In addition, SWT appears to enhance regenerative potential of injected cardiac stem cells significantly in human patients (54). Mechanistically, AKT-mediated upregulation of eNOS upon SWT induces beneficial effects on migration, proliferation, and angiogenic potential of injected cells (52). Moreover, SWT induces the release of adenosine tri-phosphate (ATP) from mesenchymal cells and, activation of purinergic receptors (55). Purinergic signaling enhances stem cell proliferation significantly. Notably, treated progenitor cells maintain multipotency *in vitro* and improve wound healing significantly by their enhanced differentiation potential (55, 56).

## Left Ventricular Remodeling

Acute myocardial infarction leads to a loss of cardiomyocytes and subsequent replacement of viable myocardium with non-contractile fibrotic scar tissue. Notably, extensive fibrosis emerging from the infarction border zone can be found as well in non-infarcted myocardium. This process of adverse left ventricular (LV) remodeling extends tissue damage, further impairs cardiac function, and ultimately worsens heart failure. LV remodeling is associated with poor prognosis and revascularization often fails to ameliorate this pathologic process. Several studies observed beneficial effects of mechanical stimulation with SWT after acute myocardial infarction. Thereby, cardiac function is preserved, possibly by limiting fibrotic remodeling of the heart (15). Effects are accompanied by angiogenesis as well as lower numbers of fibrocytes within the infarction border zone (9). Similarly, a reduced number of TGF-β positive cells is found upon SWT in a model of acute myocardial infarction in rats (57). A potential mechanism by which SWT reduces cardiac fibrosis in ischemic hearts might be through the regulation of the phosphoinositide-3-kinase (PI3K)/AKT pathway, as inhibition of PI3K abolished the observed improvement of left ventricular function and reduced cardiac fibrosis (58). Notably, similar effects are observed in myocardial ischemia/reperfusion injury (59). This model is of high clinical relevance, as SWT might be beneficial to alleviate cardiac ischemia/reperfusion injury.

## Inflammation

Upon myocardial infarction, subsequent inflammation determines the fate of the myocardium contributing to cell death, fibrosis, healing, and scar formation. Wound healing upon myocardial infarction occurs in a biphasic manner with an initial strong pro-inflammatory response followed by a prolonged resolution of inflammation, which governs tissue repair and scar formation (60). Accordingly, a balanced inflammatory response is crucial for adequate healing (61). An early proinflammatory response is necessary to remove cellular debris after ischemia, whereas the later anti-inflammatory response promotes a milieu of angiogenesis and tissue repair (62).

SWT improves myocardial function *via* modulation of the inflammatory response. SWT of endothelial cells induces the release of endogenous RNA, causing activation of innate immune receptor Toll-Like receptor 3 (TLR3) (63). This inflammatory signaling *via* TLR3 activation promotes angiogenesis after SWT in ischemic hind limbs. *In vivo*, restoration of blood flow in ischemic tissue is abolished in *Tlr3^–/–^* animals (63). TLR3 typically activates an early pro-inflammatory and a late anti-inflammatory response (64). In this manner, shockwave-induced activation of TLR3 leads to an initial release of pro-inflammatory cytokines including cyclophilin A and interleukin 6 (IL-6). With some delay after treatment, anti-inflammatory interleukin 10 (IL-10) is upregulated (65). IL-10 is a major regulator of inflammation by restricting excessive pro-inflammatory cytokine production of migrating immune cells (66). Migrating immune cells, primarily macrophages, are mainly responsible for cytokine production within ischemic tissue (67). In the tissue, macrophages polarize toward a M1 or M2 subtype. M1 macrophages maintain the inflammatory cytokine production and enhance the further recruitment of immune cells (68). M2 macrophages on the other hand suppress the immune response and resolve acute inflammation (67). Polarization toward anti-inflammatory M2 macrophages is driven again by IL-10 and SWT thereby enhances this process (69). Similar observations of enhanced M2-presence are observed in ischemic mouse hind limbs treated with SWT (70).

**FIGURE 2:**
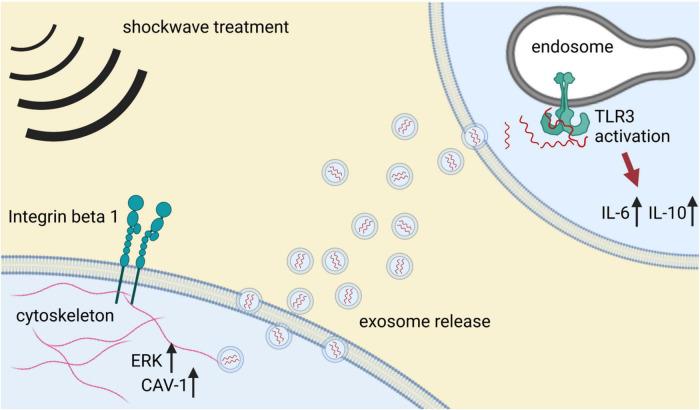
Mechanotransduction and modulation of inflammatory response upon SWT. Mechanical stimulation with shockwaves induces cellular mechanosensing. Activation of beta 1 integrin on the cell surface induces intracellular upregulation of ERK, which in turn activates caveolin 1 (CAV-1). Caveolin is a potent regulator of microvesicle release and hence, intercellular communication. Microvesicles, particularly exosomes, are loaded with specific cargo and released to the extracellular space. Uptake of shockwave-derived exosomes activates the innate immune receptor TLR3. TLR3 signaling results in an inflammatory response that may induce epigenetic alterations required for the regenerative effects of SWT.

In addition, SWT elevates NO levels *via* eNOS (51, 57) and neural NOS (71) induction and even non-enzymatic NO formation (72). Elevated NO levels increase local blood flow and thereby reduce ischemic necrosis and ensuing inflammatory processes (21). Thus, SWT reduced inflammation in a porcine model of myocardial ischemia (51). In the ischemic rat heart, it suppresses the infiltration of TGF-β positive cells and reduces the release of several pro-inflammatory cytokines while enhancing anti-inflammatory cytokines (57). Overall, these findings confirm that a modified inflammatory response mediated by TLR3 is involved in the positive effects elicited by SWT (Figure 2).

Besides the above-described mechanisms, recent research showed an emerging role of TLR3-mediated inflammation on cellular plasticity and concomitant cell fate transitions. This process, termed transflammation, may provide a mechanism by which mechanical activation of immune signaling facilitates angiogenesis in ischemic tissue.

## Transflammation

As described earlier, activation of pattern recognition receptors (such as TLR3) by cellular damage or pathogens triggers cell-autonomous inflammatory signaling that leads to the release of inflammatory cytokines and chemokines that initiate tissue inflammation. We have discovered another limb of this pathway that mediates cellular plasticity.

Specifically, we have observed that inflammatory signaling causes a global alteration in the expression and activity of epigenetic modifiers. For example, activation of TLR3 by retroviral RNA increases the expression of histone acetyltransferases (HATs) and reduces the expression of histone deacetylases (HDAC). This change in the balance of epigenetic enzymes favors histone acetylation and thereby an open chromatin state, which can facilitate cell fate transitions (73). Furthermore, inflammation leads to nuclear translocation of inducible nitric oxide synthase (iNOS). There, it binds to, and S-nitrosylates epigenetic modifiers such as elements of the polycomb repressive complex (PRC1) and the NURD complex, causing these suppressive epigenetic enzymes to dissociate from the chromatin, enabling access to previously repressed transcriptional programs (74, 75). Finally, this inflammatory pathway activates a glycolytic switch, which supplies more citric acid to the nucleus, where it is converted to Acetyl-CoA to facilitate histone modifications (76).

This process of transflammation is required for changes in somatic cell fate, such as that which occurs when a fibroblast is reprogrammed to an induced pluripotent stem cell, or to an endothelial cell (73, 77, 78). Furthermore, transflammation appears to be activated in the setting of ischemia and may play a role in perfusion recovery. Specifically, we have observed a role for transflammation in the transdifferentiation of resident fibroblasts to endothelial cells in recovery of limb ischemia. Anti-inflammatory agents impair the transdifferentiation of fibroblasts to endothelial cells, impair perfusion recovery, and exacerbate tissue necrosis in a murine model of limb ischemia (79).

Since the underlying mechanism of this regenerative process is a modest activation of inflammatory signaling, as observed after shockwave therapy, further research should be done to clarify if mechanical conditioning could potentially have its effect on therapeutic cell fate transitions.

## Mechanotransduction

The beneficial effects of SWT were initially thought to be due to mechanical, non-selective tissue damage followed by repair mechanisms. However, more recent work indicates that SWT induces a specific tissue response. How the physical stimulus of SWT is translated into a specific biological response is beginning to be elucidated. Cells are equipped with mechanosensors responsible for the translation of mechanical input to a biological response, a process termed “mechanotransduction” (80). Integrins play a major role within the process of mechanosensing. Integrins are cell surface receptors binding proteins of the extracellular matrix (80). They are linked intracellularly to actin filaments of the cytoskeleton, initiating their reorganization, and transducing molecular mechanism among others *via* AKT/ERK activation. Mechanical stimulation of cells with shockwaves induces this particular integrin-mediated AKT/ERK signaling (81). Besides activation of cellular mechanosensors, the cellular membrane itself is highly responsible to mechanical stimulation. Under the influence of SWT, the membrane can release vesicles from its surface. These reactive mechanisms rely on expression of caveolin 1 (CAV-1), which is upregulated upon SWT (81). CAV-1 governs the release of microvesicles, an important component of intercellular communication (82).

These observations are consistent with findings that SWT induces paracrine effects, as transfer of supernatant from SWT-treated cells recapitulates the direct effect of SWT. Treated supernatants contained increased amounts of released growth factors, protein/RNA complexes as well as exosomes (26, 83, 84). These specific extracellular vesicles are derived from cytosolic multivesicular bodies upon treatment and show distinct angiogenic potential *in vitro* as well as *in vivo*. Shockwave-derived exosomes improve vascularization and cardiac function in ischemic hearts. Of interest, inhibition of exosome release abolished the angiogenic effects of SWT. Intriguingly, shockwave-derived exosomes differ from control exosomes by their cargo. The angiogenic microRNA miR19a-3p mediates angiogenesis and reduction of myocardial fibrosis upon SWT (83). Use of miR19a-3p obtained the same results as shockwave-derived exosomes, whereas antagonizing this specific miRNA abolished the angiogenic potential of SWT exosomes. Further studies are needed to elucidate the exact mechanisms of extracellular vesicle release upon SWT and their potential interplay with innate immunity (Figure 2).

## Discussion and Perspective

Ischemic heart disease and ischemic heart failure are ever increasing in the western world. Together they are a leading cause of death and disability, representing a major socio-economic burden for healthcare systems (1). Current treatment strategies fail to regenerate damaged heart muscle. Cell-based regenerative options have been disappointing (85). However, small single-center studies suggest that SWT may be useful in patients with ICMP (5). However, most clinical studies of cardiac SWT used symptomatic relief as a primary endpoint rather than objective improvement in heart function. Moreover, all available clinical data was generated by extracorporeal application of SWT. Extracorporeal application of SWT to the ischemic heart has several limitations: (a) a small acoustic window, (b) accessible treatment regions being restricted to the anterior myocardium, and (c) the risk of potential lung injuries (86, 87). Hence, a direct epicardial approach during surgical procedures may be more favorable to obtain optimal treatment efficacy since beneficial effects are directly associated with the intensity of mechanical stimulation (24). A new clinical trial of direct epicardial SWT in patients with ICMP undergoing coronary artery bypass grafting aims to determine if direct application of SWT to the myocardium can increase cardiac function (88).

To conclude, effects of shockwaves have been studied extensively in ischemic tissue, including the ischemic heart. Thereby, it is application has been tested in models of both acute and chronic myocardial ischemia (23, 27, 58). In both settings SWT showed positive effects on cardiac function, although clinical settings are mainly focused on chronic IHD. SWT induces various molecular mechanisms leading to the release of angiogenic growth factors, enhanced survival of hypoxic cells and regenerative epigenetic mechanisms *via* induction of inflammatory signaling. Underlying these observed effects is the process of mechanotransduction, the translation of a mechanic stimulus to a biological signal. The cell membrane is highly responsive to shockwaves and sheds extracellular vesicles upon treatment. These vesicles have angiogenic activity and are capable of improving vascularization in ischemic tissue (83). The effect of SWT to induce angiogenesis may not fully explain the observed improvement of LV remodeling. Although angiogenesis is the most prominent factor in regenerating chronic ischemic tissue, mechanical conditioning also seems to have a protective role *via* anti-apoptotic effects. Both effects are accompanied by reduction of cardiac fibrosis, either by preventing its initial formation or by degradation of fibrotic material when tissue perfusion is restored. Further research should show whether mechanical stimulation *via* shockwaves may induce cardiac-specific mechanisms in comparison to other soft tissue applications. Furthermore, it remains to be clarified which cell types within the ischemic heart are primarily responding to the mechanical stimulation with shockwaves since different cell types showed varying effects upon treatment *in vitro* (24). In addition, further in-depth analysis of the SWT-induced release of exosomes and their cargo is required to provide more comprehensive insight how this may interplay with or activate other crucial mechanisms such as the inflammatory response. Although the molecular mechanisms are incompletely characterized, evidence is accumulating that SWT has beneficial effects in patients suffering from myocardial ischemia. Notably, existing data is restricted to small observational monocentric studies with limitations regarding variations in extent of myocardial injury, treatment protocols and endpoint analyses. Therefore, multi-center adequately powered randomized double-blind studies are warranted to assess the safety and efficacy of SWT in IHD.

## Author Contributions

MGa: conceptualization, writing original draft, and writing review and editing. FN, JHi, LP, VS, MGi, and JC: review and editing. CG-T and JHo: conceptualization, supervision, and writing review and editing. All authors contributed to the article and approved the submitted version.

## Conflict of Interest

JHo, MGi, and JC were shareholders of Heart Regeneration Technologies GmbH, an Innsbruck Medical University spin-off aiming to develop cardiac shockwave therapy (www.heart-regeneration.com). The remaining authors declare that the research was conducted in the absence of any commercial or financial relationships that could be construed as a potential conflict of interest.

## Publisher’s Note

All claims expressed in this article are solely those of the authors and do not necessarily represent those of their affiliated organizations, or those of the publisher, the editors and the reviewers. Any product that may be evaluated in this article, or claim that may be made by its manufacturer, is not guaranteed or endorsed by the publisher.
